# Water Nitrate Remote Monitoring System with Self-Diagnostic Function for Ion-Selective Electrodes

**DOI:** 10.3390/s21082703

**Published:** 2021-04-12

**Authors:** Dae-Hyun Jung, Hak-Jin Kim, Joon Yong Kim, Soo Hyun Park, Woo Jae Cho

**Affiliations:** 1 Department of Biosystems and Biomaterial Engineering, College of Agriculture and Life Sciences, Seoul National University, Seoul 08826, Korea; jeoguss@gmail.com; 2 Smart Farm Research Center, Korea Institute of Science and Technology (KIST), Gangneung-si 25451, Korea; ecoloves@kist.re.kr; 3 Research Institute of Agriculture and Life Sciences, Seoul National University, Seoul 08826, Korea; tombraid@snu.ac.kr (J.Y.K.); woojae56@gnu.ac.kr (W.J.C.); 4 BK21 Global Smart Farm Educational Research Center, Seoul National University, Seoul 08826, Korea; 5 Division of Agro-System Engineering, College of Agriculture and Life Science, Gyeongsang National University, Jinju 52828, Korea

**Keywords:** online monitoring, ion-selective electrode, self-diagnostic method, MQTT

## Abstract

The detection of nitrate pollutants is a widely used strategy for protecting water sources. Although ion-selective electrodes (ISEs) have been considered for the determination of ion concentrations in water, the accuracy of ISE technology decreases owing to the signal drift and decreasing sensitivity over time. The objectives of the present study were: (1) to develop an online water monitoring system mainly consisting of an Arduino board-based Internet-of-Things (IoT) device and nitrate ISEs; and (2) to propose a self-diagnostic function for monitoring and reporting the condition of the ISEs. The developed system communicates with the cloud server by using the message queuing telemetry transport (MQTT) protocol and provides monitoring information through the developed cloud-based webpage. In addition, the online monitoring system provides information on the electrode status, which is determined based on a self-diagnostic index (SDI, with a range of 0–100) of the electrode drift and sensitivity. The diagnostic method for monitoring and reporting the electrode status was validated in a one-month-long laboratory test followed by a field test in a stream near an agricultural facility. Moreover, a self-diagnostic index (SDI) was applied in the final field experiments with an accuracy of 0.77.

## 1. Introduction

In many parts of the world, rapid industrialization and population growth in urban areas have increased the water demand and gradually worsened the water quality owing to the depletion of the water volumes in rivers. The dams and reservoirs, which have been constructed to retain and manage such water resources efficiently, slow down the cycling of the water volumes in the river systems; hence, the pollutants cannot be fully removed, and the water quality degenerates. In particular, the excessive inflow of nutrient salts such as nitrates and phosphates causes multiple problems such as eutrophication, which results in the growth of algal blooms and reduced water availability. Hence, managing these pollutants requires special government-driven management programs [1,2].

Monitoring systems for aquatic environments play a crucial role in various water uses such as human usage, aquaculture, livestock watering, irrigation, and agricultural fertigation. The detection of nitrate pollutants in water has been widely adopted to protect water sources. Nitrates are common groundwater contaminants that can pose serious health risks because their colorless, odorless, and tasteless properties render them difficult to detect by the human senses [3,4]. Therefore, much effort has been devoted to reducing pollution via the precise monitoring of water and wastewater in the agricultural field. In addition, accurately measuring ionic concentrations can provide useful information on the biogeochemistry of aquatic ecosystems [5].

Ion-selective electrodes (ISEs) have been used for the determination of ion concentrations in a wide range of clinical, environmental, and food analysis applications, thereby extending the application range of potentiometry. The successful application of ISE technology in real-time sensing of nitrate requires the continuous measurement of individual nutrient concentrations with acceptable sensitivity [5,6]. However, one of the major deficiencies of ISE technology, compared to standard spectroscopy-based analyzers, is the potentially limited measurement accuracy due to the signal drift and loss of sensitivity over time [7]. Indeed, the signal drift can be the primary factor affecting the determination accuracy for nutrient concentrations when an ISE-based on-site ion monitoring system that requires frequent immersion of the electrodes into a solution is used [8]. The reasons for the signal drift are very complex; it is typically caused by a change in the membrane of an electrode and leaching of the inner solution. The contact between the membrane and electrode body may become obscured owing to the deposition of organic matter; in addition, the contact may be lost owing to surface wear caused by mechanical stress; these are the main causes of changes in the membrane [9]. Therefore, a sensor must be replaced when it shows a decrease in performance or loses its function, which can result in data loss. However, the previously mentioned factors make it difficult to predict the status of the electrode and hence the replacement time on site.

Researchers are actively studying sensor-based monitoring technology for diagnosing the state of other systems or for monitoring system errors [10,11]. According to relevant reports, the most important aspect is to develop an appropriate algorithm for fault detection. In turn, the majority of studies of fault detection algorithms have adopted methods that incorporate a diagnostic model [12]. Bertrand–Krajewski et al. Referenxe [13] proposed a sensor for an uncertainty index and applied a law of propagation of uncertainties based on a 1st-order Taylor series function to a sensor diagnosis system. Ramanathan et al. [14] presented five rule-based fault detection methods for evaluating the ISE performance and demonstrated that it can be calibrated to diagnose faults. In addition, Jung et al. [6] and Cho et al. [15] proposed a two-point normalization method for correcting the sensitivity change and signal drift during the long-term use of an array of multiple ISEs. Moreover, the potential advantages of using AI-based techniques in condition monitoring and diagnosis have been widely reported [16,17].

Water quality monitoring systems with ISEs are advantageous for long-distance communication. In particular, a communication system that exchanges information to respond immediately to sensor failures on-site is required. Many researchers have reported that the previously mentioned diagnostic systems create synergies when combined with Internet-of-Things (IoT) technology; in addition, IoT has been applied in many recent sensing technology studies [18,19,20]. These technologies primarily form part of a system such as an automatic on-site water quality measurement, a data logging, and wireless communication system. This system type is particularly useful because it allows the user to monitor the conditions of the data and system at any time and in any place [21,22,23]. Recently, an intelligent diagnostic algorithm for discriminating abnormal power supply in IoT-based buildings was proposed [24]. In addition, a deep learning-based approach as an intelligent fault diagnosis algorithm has been recently attempted in image-based systems, and a high probability has been reported [25]. Despite their extensive use and great success, the focus is on the analysis of environment field monitoring data, and these diagnostic algorithms rarely applied to the water quality monitoring system. Thus, if IoT technology is used to monitor continuously the environmental information and condition of the measuring instruments and sensors, any sudden degradation of the ion sensor can be effectively managed.

In this study, an IoT-based nitrate monitoring system was developed and evaluated in an on-site application for stable water quality monitoring. In addition, the results of a laboratory test and past performance were used to propose a self-diagnostic index (SDI) for the detection of faults of the nitrate measurement sensor. The developed system reacts to sudden performance problems and notifies the user of sensor failures in real time; thus, data loss can be prevented, and the reliability of the monitoring system is ensured.

## 2. Materials and Methods

### 2.1. Configuration of IoT-Based Nitrate Measurement System

The sensor was designed based on the materials and method proposed by previous research [5,6]. A polyvinyl chloride-based membrane was used to fabricate a nitrate measurement electrode. The membrane was prepared using the compositions of tetradodecylammonium nitrate (TDDA), 2-nitrophenyl octylether (NPOE), and high-molecular-weight polyvinyl chloride (PVC), as shown in the Table 1. The NO_3_^−−^ ISEs were filled with an internal solution of 0.01 M NaNO_3_ + 0.01 M NaCl. An Ag/AgCl electrode prepared by coating silver wire (99%) with a diameter of 1 mm with Ag/AgCl ink (model 01164, ALS Co., Tokyo, Japan) was immersed as the inner reference electrode. A double junction electrode (Orion 90-02, Thermo Fisher, Waltham, Mass.) was used as the reference electrode.

**Figure 1:**
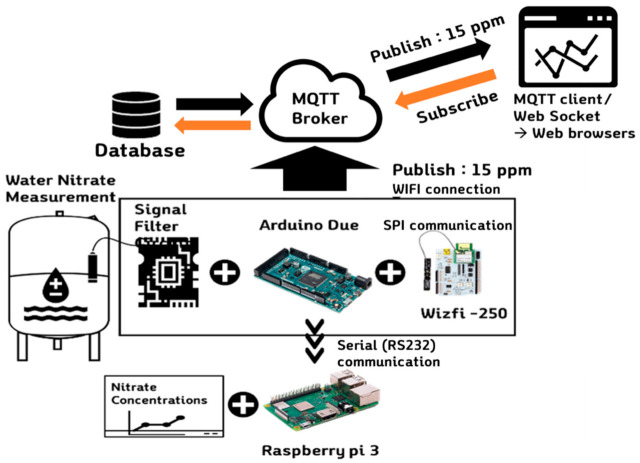
Configuration of Internet-of-Things (IoT)-based online water quality measurement system.

In addition, an automatic system was configured to incorporate a metering pump (SR10/30, ASF THOMAS, Germany) and a sampling pump (SR10/60, ASF THOMAS, Fürstenfeldbruck, Germany) for the measurement of two standard solutions; a valve was integrated to exhaust the samples. As shown in Figure 1, the proposed system was fully configured with an open source-based IoT system. An Arduino Due board (Arduino LLC, MA, USA) was used as a low-cost microcontroller to log the signals of the electrode and deliver messages to a server via a WizFi-250 module (WIZnet Co., Ltd., Seongnam, Korea) for wireless communication. The Arduino Due has a driving voltage of 3.3 VDC and an analog-to-digital converter (ADC) resolution of 12 bits; it is suitable for multiple-channel analog collection and digital signal processing. In the previous study [5], a water quality monitoring mainboard, an ADC chip, and a relay were connected to a filter through a jump line and a breadboard; however, the signal stability was affected by measurement noise. Accordingly, a printed circuit board (PCB) to which the signal filter, Arduino Due board, and WizFi module were attached and six additional relays were installed to facilitate the on/off control of the monitoring system. The Arduino board connects WIFI to the WizFi module through SPI communication, publishes MQTT messages, and then transmits raw data to the Raspberry Pi board through RS232 serial communication to store the data separately.

The developed system communicates with the cloud server by using the message queuing telemetry transport (MQTT) protocol and provides monitoring information through the developed web-based monitoring page. A web-based nitrate monitoring page was constructed and tested to monitor measurement data on the web. As shown in Figure 1, the message queuing telemetry transport (MQTT) protocol was implemented through the Arduino Due-embedded board, thereby enabling it to recognize the broker server via the wireless network and to publish data from the three nitrate electrodes [26]. Moreover, the web-based nitrate monitoring page was constructed and tested to monitor measurement data on the web. The data are published on the broker’s own webpage and sent on to the user. The web page was hosted using a commercial cloud server (Naver Cloud Platform, Naver, Seongnam, Korea) and the server configuration was Node.JS, and Vue.JS was used for building a front-end UI.

**Figure 2:**
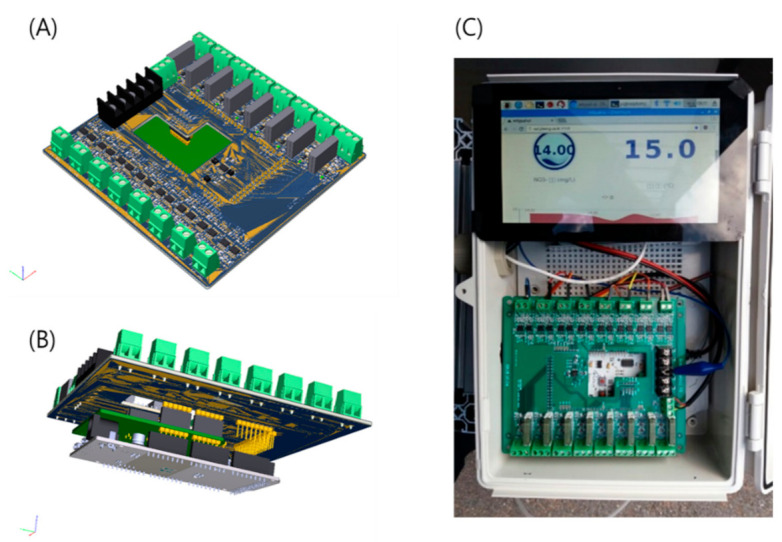
Three-dimensional schematic of the proposed printed circuit board (PCB): (**A**) upper part, (**B**) lower part; (**C**) photograph of installed PCB.

**Figure 3:**
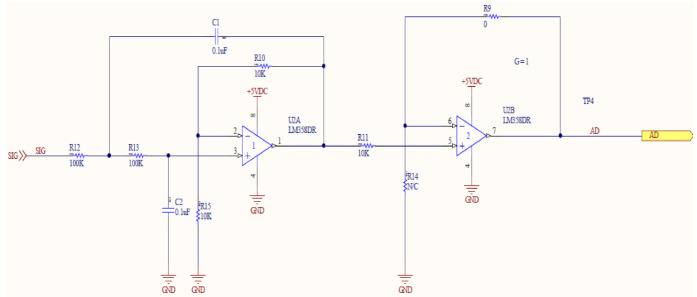
Signal-processing filter for ISE.

The upper and lower parts of the PCB are schematically shown in Figure 2A,B, respectively. The Arduino board and WizFi module were connected to the lower part to realize the installation in Figure 2C. The PCB signal was filtered with a Sallen–Key second-order filter to collect stably the electrode signals (Figure 3). This filter has a 10.33 Hz low-pass cutoff frequency; it can amplify signals twice and remove inter-channel disturbance. To display the current concentrations on the LCD panel, a Raspberry Pi board (Raspberry Pi Foundation, UK) was added to the monitoring system. The Raspberry Pi board was installed to check the LCD screen in the field, and additionally, in preparation for problems with internet connection, raw data was stored using it as a backup DB. In addition, a separate database (DB) was constructed to collect raw data, and a self-diagnostic algorithm for the electrodes (explained below) was installed for calculation.

**Figure 4:**
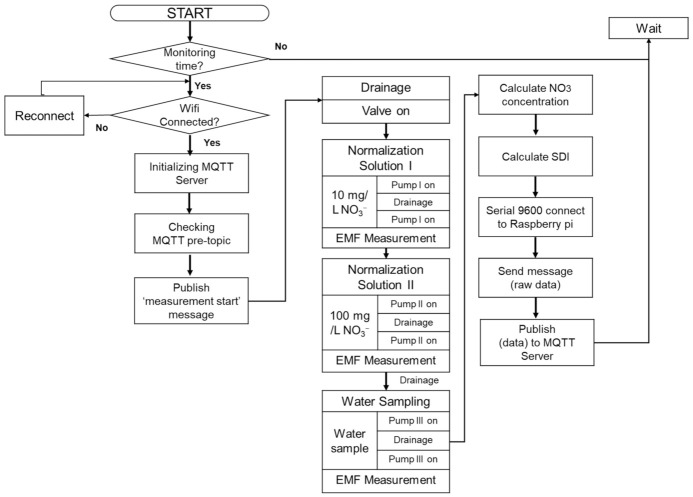
Flow chart of sequence of online water monitoring system.

The overall sequence of the developed water nitrate monitoring system with self-diagnostic function is shown in Figure 4. The sequence includes a two-point normalization setup with rinsing, sampling pumps, drainage via a solenoid valve, and measurements with the ISEs, while the data communication processes with the MQTT server and fault diagnosis are presented. The two-point normalization method consisting of a sensitivity adjustment followed by an offset adjustment was used to minimize the potential drift. Two different concentrations of 10 and 100 mg L^−1^ were used as known standard solutions of low and high concentrations, respectively, to determine the slope and offset values prior to sample measurement. Through this process, the signal drift, sensitivity, and deviation values were obtained for each electrode required for the self-test function.

### 2.2. Self-Diagnostic Algorithm for ISE

#### Selection of SDI for Electrode

The change in sensitivity is an essential factor for assessing the performance of the ISE [14]. In addition, the continuous data from sensor signals must be analyzed to determine whether there is a signal drift (which is also an important index for assessing the sensor performance). In this study, the current status of the nitrate electrodes was quantitatively checked using three indicators, i.e., electrode drift index (S_1_), sensitivity change index (S_2_), and estimated value change index between multiple electrodes (S_3_):

(1) $$S_{1}\left(\%\right)=\frac{\left|EMF_{\left(n\right)}-EMF_{\left(n-1\right)}\right|+\left|EMF_{\left(n\right)}-EMF_{\left(n-2\right)}\right|+\left|EMF_{\left(n\right)}-EMF_{\left(n-3\right)}\right|}{3\cdot EMF_{n}}\times 100$$

(2) $$S_{2}\left(\%\right)=\frac{\left|Sensitivity_{initial}-Sensitivity_{current}\right|}{Sensitivity_{initial}}\times 100$$

(3) $$S_{3}\left(\%\right)=\frac{\left|\bar{Expected\;value}-Expected\;value\left(i\right)\right|}{\bar{Expected\;value}}\times 100$$

The SDI (%) is presented in Equation (4):

(4) $$\left(\mathrm{SDI}\right)=S_{1}+S_{2}+S_{3}$$

The drift index (S_1_, Equation (1)) represents the drift from the standard electromotive force (in mV) of the ISE, indicating a change in *EMF* values determined via three identical and consecutive measurements of a standard solution before the use of the electrode in the practical measurements. The sensitivity index (S_2_, Equation (2)) reflects the change in the sensitivity of the electrode (in mV) due to the presence of foreign substances or bubbles on the membrane surface; it is determined with standard NO_3_^−^ solutions of two concentrations (i.e., 10 and 100 ppm (mg L^−1^)) before the practical measurements. Finally, the deviation from other electrodes introduced when predicting the concentration of a sample is represented by S_3_ (Equation (3)). In this study, it is the difference between the average prediction result of multiple electrodes and each individual prediction. A laboratory test was performed to select appropriate values for each index to determine the replacement time of the electrode.

### 2.3. Experimental Procedure

#### 2.3.1. Laboratory Test for Effectiveness of Self-Diagnostic Algorithm

Before the field installation of the developed online water quality measurement system, the performance of the system and effectiveness of the self-diagnostic algorithm were evaluated in a laboratory test in which samples of tap water from Seoul were repeatedly tested at 8 h intervals for one month. Before each series of measurements, two standard NO_3_^−^ solutions with different concentrations (10 and 100 ppm (mg L^−1^)) were used to determine the sensitivity and drift of the electrode with respect to the accurate nitrate concentrations of the samples identified by a commercial soil and water quality analysis center (National Instrumentation for Environmental Management (NICEM), Seoul, Korea).

#### 2.3.2. Field Verification Experiment

**Figure 5:**
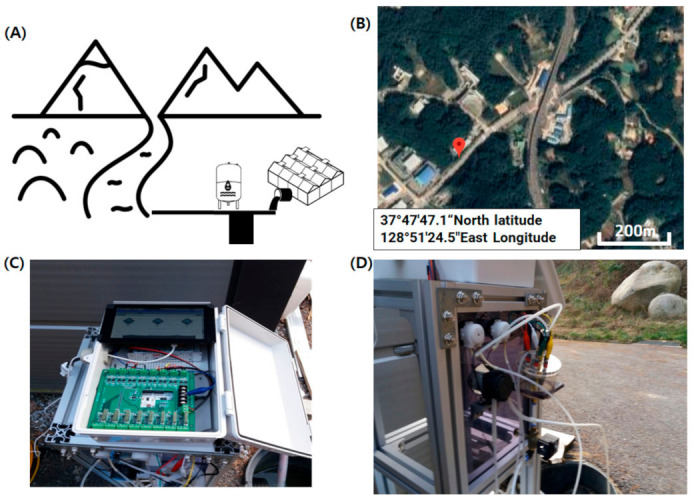
Field-test site and configuration of monitoring equipment: (**A**) schematic diagram of test location; (**B**) satellite image showing location latitude and longitude; (**C**) online stationary monitoring system, and (**D**) array of three nitrate ISEs.

As shown in Figure 5, a field monitoring experiment was conducted to operate and evaluate the self-diagnostic functions of the developed online water nitrate monitoring system and ISE. The test site is schematically shown in Figure 5A; the water source is indicated by the nearby mountainous area. The location (37°47′47.1″ North and 128°51′24.5″ East) is presented in the satellite image in Figure 5B. In addition, because the water quality of the test site was expected to change owing to waste water emissions from the nearby residential and agricultural areas, the developed online stationary monitoring system was used to monitor the nitrate concentration (Figure 5C). The monitoring experiments were conducted four times at 6 h intervals each day; during each experiment, 16 samples were collected and sent to the NICEM laboratory to determine actual concentrations in the samples. The three nitrate ISEs were installed in an array (Figure 5D), and their mean value was sent to the online webpage. Subsequently, the self-diagnostic algorithm identified the replacement time corresponding to the electrode values, which was determined with the index that had been validated in the previously mentioned laboratory test.

#### 2.3.3. Evaluation of Self-Diagnostic Methods

The precision, recall, and accuracy of the setup were investigated in this study to determine the effectiveness of the SDI. The factors that evaluate a model can eventually be defined as the relationship between the model’s predicted and actual correct label. The result is classified into “True and False”; thus, the classification model outputs “True” and “False”, thereby dividing the results into a 2 × 2 matrix (Table 2) [27].

The precision (*π_i_*) is determined as the conditional probability that represents the classifier’s ability to place a value into the correct category as opposed to placing all values (correct or incorrect) into the same category:

(5) $$\pi_{i}=\frac{TP_{i}}{TP_{i}+FP_{i}}.$$

The recall (*ρ_i_*) is defined as the probability that, if the model should be assigned the label “True” under the actual category in True, the result is considered “true positive”. The recall is presented in Equation (6):

(6) $$\rho_{i}=\frac{TP_{i}}{TP_{i}+FN_{i}}.$$

Although accuracy is commonly used to evaluate categorization techniques, this measure is much less sensitive to variations in the number of correct decisions than precision and recall. The accuracy ($\alpha_{i}$) is defined as follows:

(7) $$\alpha_{i}=\frac{TP_{i}+TN_{i}}{TP_{i}+TN_{i}+FP_{i}+FN_{i}}$$

Thus, the electrode condition was classified into three states (normal, management, and replacement); “replacement” and “management” were categorized as “True”, and “normal” was categorized as “False”.

## 3. Results

### 3.1. Lab Test Results Following Application of SDI

**Figure 6:**
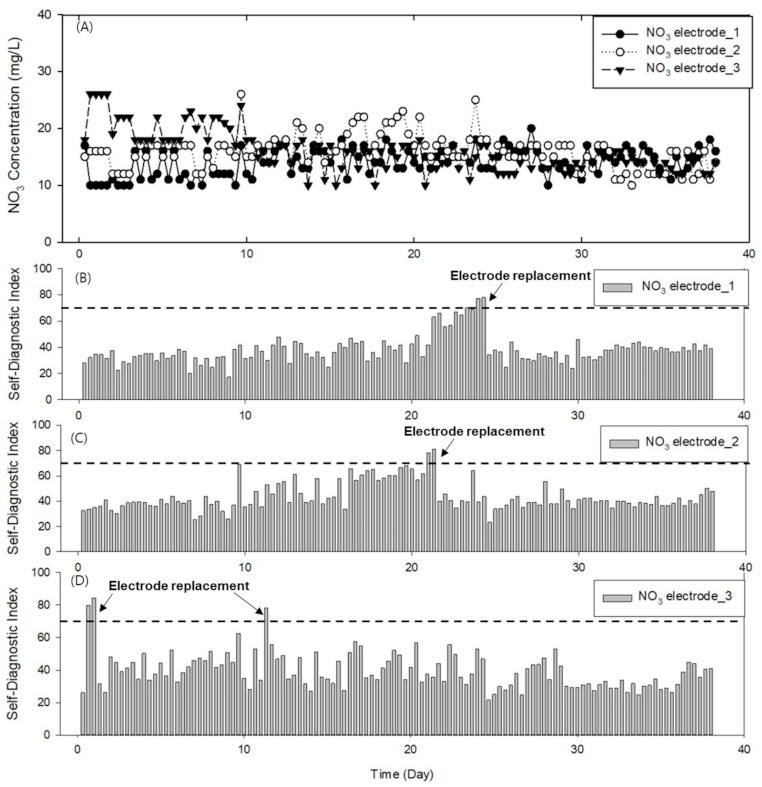
Results of monitoring NO_3_^−^ concentration in laboratory setting: (**A**) NO_3_^−^ concentrations provided by each electrode; (**B**–**D**) changes in SDI and electrode replacement histories of (**B**) electrode 1, (**C**) electrode 2, and (**D**) electrode 3.

**Figure 7:**
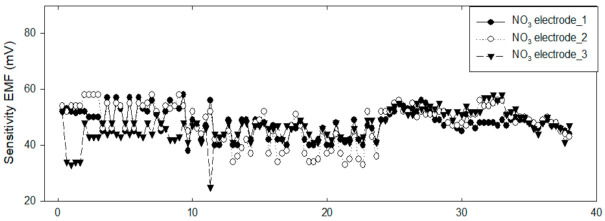
Sensitivity variation of three NO_3_^−^ electrodes during laboratory test.

The effectiveness of the developed online monitoring system and self-diagnostic algorithm was tested by continuously measuring a sample in the laboratory setting for three days. The repeated measurements of the samples with an NO_3_^−^ concentration of approximately 17.85 mg/L according to the standard analysis resulted in an average NO_3_^−^ concentration of 15.51 ± 3.38 mg/L, which is similar to that of the real samples. The results are presented in Figure 6; the predicted NO_3_^−^ concentrations of each electrode are plotted in Figure 6A, and the corresponding SDIs in Figure 6B–D are the SDIs of each electrode. In the test, an SDI of 70 or higher indicated the optimal time for the replacement of the electrode. In the case of NO_3_^−^ electrode 1 (filled circles, Figure 6A and bar chart in Figure 6B), the SDI exceeded 60 on Day 21 and 70 on Day 24, at which point the electrode was replaced. In the case of NO_3_^−^ electrode 2 (open circles, Figure 6A and bar chart in Figure 6C), the SDI reached 68 on Day 9. On that day, the bubbles on the electrode membrane were removed by the experimenter, and the SDI recovered to its original value. On (approximately) Day 13, the electrode showed a decrease in its sensitivity and a change in the drift, as shown in Figure 7 (open circles). On (approximately) Day 22, the electrode 2 was replaced because the SDI was approximately 79. By contrast, the NO_3_^−^ electrode 3 (filled triangles, Figure 6A and bar chart in Figure 6D) exhibited a sensitivity of approximately 37 on Day 1, which indicates a change in the sensitivity of the membrane compared to those of the other electrodes (Figure 7). Consequently, the electrode was replaced when the SDI exceeded 75. The electrode sensitivity momentarily decreased 11 days after the replacement. Accordingly, the electrode was replaced again.

These laboratory test results show that the electrodes can be replaced on time based on the proposed implemented self-diagnostic algorithm. Consequently, nitrate ions can be continuously and effectively monitored.

### 3.2. Learning Results of Deep Neural Network-Based Diagnostic Model for Electrode Status

**Figure 8:**
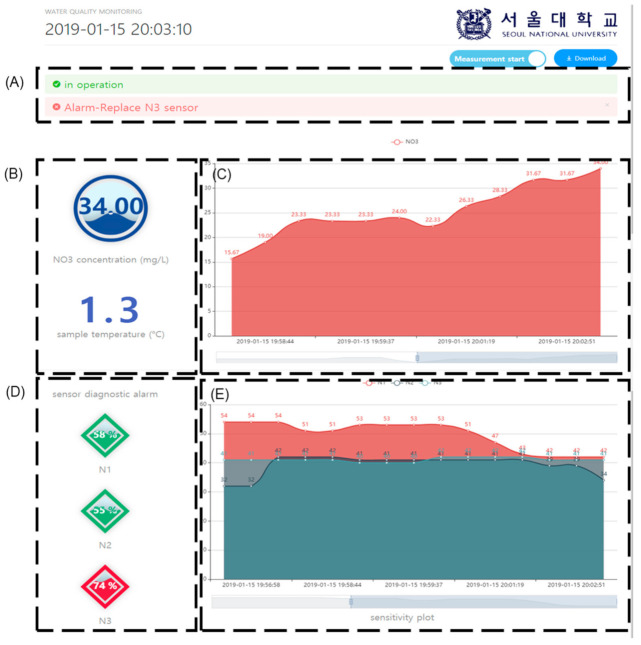
Cloud-based water quality monitoring webpage developed in this study: (**A**) operative status of system (green) and electrode replacement alarm (red); (**B**) current nitrate concentration and representative temperature of water resource; (**C**) plot of nitrate concentration and point data; (**D**) self-diagnostic indices (SDIs) of electrodes and (**E**) previous sensitivity data.

As shown in Figure 8, the web-based nitrate monitoring page was constructed and tested to monitor the measurement data and provide the following three types of information: (i) the current nitrate concentration (Figure 8B) was monitored and plotted as a continuous graph over time (Figure 8C); (ii) the change in the sensitivity of each electrode in Figure 8E indicates the SDI (Figure 8D); (iii) based on this value, an alarm message was generated when the electrode had to be replaced (Figure 8A). In Figure 8C, a relatively rapid change in NO_3_^−^ concentration is observed for a short period of time, which might be affected by the detection of an abnormality in the N3 electrode.

### 3.3. Field Verification Experiment

**Figure 9:**
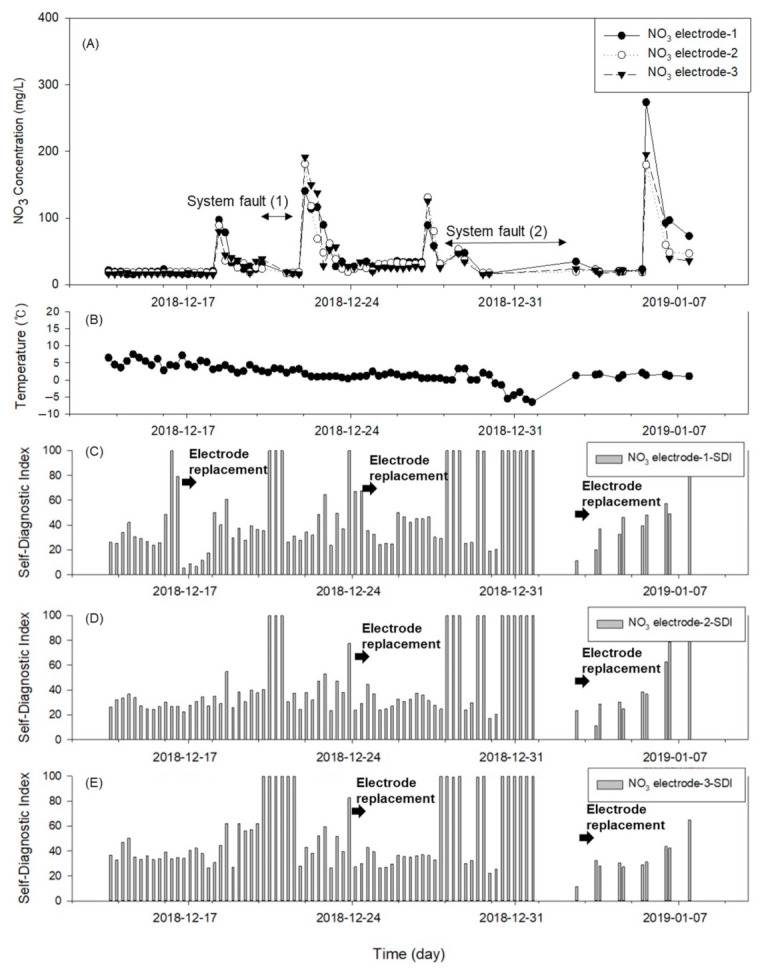
Field monitoring results: (**A**) NO_3_^−^ concentration; (**B**) temperature variation; (**C**–**E**) variation in SDI and replacement histories of (**C**) electrode 1, (**D**) electrode 2, and (**E**) electrode 3.

**Figure 10:**
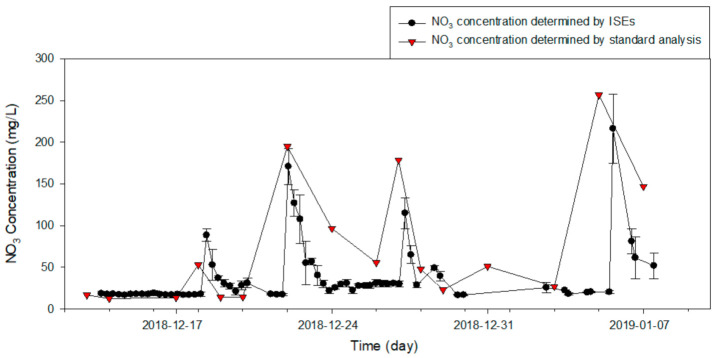
Comparison of nitrate concentrations predicted by ISE and standard analyzer.

The application results of the developed online monitoring system and self-diagnostic algorithm for the on-site determination of the electrode status are presented in Figure 9. The variation in the measured nitrate concentration of each electrode is plotted in Figure 9A, and their average is compared with the concentration graph obtained from the standard analysis of real samples in Figure 10. As shown in Figure 9C–E, the SDI developed in the present study advised the user to replace an electrode during the field experiment; ten alerts were issued (excluding redundancy), and seven electrodes were replaced. Among the ten alerts, two were generated because of an external factor rather than a problem with the electrode itself. The external factor that includes an ambient temperature below zero might affect a system malfunction. The times of these erroneous alerts are indicated in Figure 9A. During system fault (1), the prepared standard solution with the lower concentration was exhausted, which resulted in an erroneous fault prediction. During system fault (2), the temperature of the tank was between 0 °C and −7 °C according to Figure 9B. Hence, it was assumed that the measurement setup was frozen. Consequently, the nighttime measurements were not conducted, and the daytime measurements were conducted twice to collect sufficient data. During those times, the SDI sent warnings about abnormally working electrodes to the user who immediately replaced the respective electrodes. The nitrate concentration significantly increased to values between 150 and 300 mg/L over an interval of three to five days before returning to its original level (20 mg/L). Because the real standard analysis results in Figure 10 reveal similar trends at the same time point, the concentration data collected by the proposed monitoring system excellently reflect the change in the water quality.

In this field experiment, the verification results of the electrode replacement timing and the classification of the abnormal state of the actual electrode through SDI can be confirmed through Table 3. The overall classification accuracy was 0.77, and the recall for determining the actual replacement time was 0.87. However, although the SDI algorithm predicted that the ISE was an actual replacement, there were many cases where the electrode state was not in the replacement state, therefore the precision was 0.67, which was lower than other indicators.

**Figure 11:**
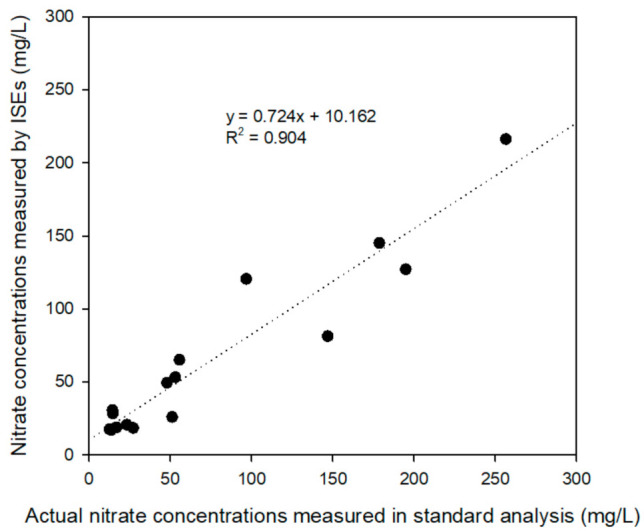
Linear relationship between ion concentration determined by ISEs and standard analyzers.

The collected samples were determined as actual concentrations using ion chromatography as a standard analysis method. The linear relationship between the ion concentrations determined by the ISEs and standard analyzers is presented in Figure 11; the curve has a slope of 0.724 and an offset of 10.162. This indicates that the two sets of measurements are similar; nevertheless, the ISE measurement results tend to be lower than the actual concentrations. Furthermore, the high coefficient of determination (R^2^) of 0.904 indicates that the performance of the electrodes can be improved via compensation.

In summary, the laboratory and field results indicate that the electrodes could be used for approximately 20 days without replacement in the laboratory test; the field monitoring experiment resulted in more frequent replacements. This was attributed to the climate conditions in winter; for example, the periodic freezing of the electrode surface adversely affected the durability of the membrane. Accordingly, the electrode status reacted sensitively to the temperature, which indicates the importance of a self-diagnostic algorithm for the determination of the electrode status.

## 4. Discussion

Although ISEs have received great attention as water quality measurement devices owing to their fast reaction and user friendliness, many researchers have reported frequent failures in field applications [28,29,30]. When the measurement target is not sufficiently accessible, the sudden failure of the ISE cannot be dealt with immediately, which may result in the loss of effective data and problems in the water quality management owing to false information. This paper proposes an algorithm that diagnoses the status of ISEs, displays the replacement time, and verifies its own effectiveness. It has been reported that an error in the measurement system may occur due to changes in external temperature or changes in the electrode itself when the actual ISE electrode is applied in the field [6,7]. The proposed SDI is an empirical index that reflects electrode drift, sensitivity change, and precision of expected values and obtained 77% accuracy for electrode conditions as a result of field experiments. In addition, the recall for the probability of an electrode malfunction was about 87%, which was used as an indicator to deliver an effective alarm. In addition, the two-point normalization method was applied to record the SDI index and to compensate for changes in the sensitivity of the electrode. It could additionally be expected to reduce the temperature change that affects the potentiometric measurements with ISEs.

In this study, an online wireless nitrate monitoring system was installed at a water supply source, and the variations in the concentration were monitored online in real time. Despite the sudden changes in the concentration, highly concentrated reagents could be accurately measured to provide the user with accurate information. According to the results of the field experiment, the measured region exhibited drastic increases in the concentration at two- to three-day intervals owing to the discharge of waste water from a nearby agricultural facility. This facility uses a hydroponic system that supplies nutrients in the form of an aqueous solution. When the amount of nutrient solution reaches a certain level, the waste nutrient solution is automatically exhausted through a drain. In particular, although the facility has a primary nitrate purifier, the developed monitoring system demonstrated that the waste nutrient solution can be one of the major causes of water pollution in this region. In Europe, because of the Nitrates Directive, the nitrate levels in water have been decreasing since 1990 [31], and the emission of nutrient solutions from agricultural facilities has been further restricted. In addition, researchers have tried to optimize the management of nutrient solutions to mitigate environmental pollution [32]. Although no relevant policies have been implemented in South Korea, the awareness of water pollution due to green algae has prompted public agencies and authorities to establish suitable measures for managing the nitrate concentrations in water. Against this background, the online nitrate monitoring system developed in this study is a useful tool for many application fields.

In addition, an algorithm for diagnosing the status of nitrate electrodes was proposed and used in a field experiment to operate electrodes. A more intuitive method for identifying and indicating the electrode replacement time was established with the help of SDIs, which gradually increased up to the proposed threshold (SDI ≥ 70). Accordingly, SDIs can be good indicators for determining the status of an electrode before its failure. There were two system faults during the field experiment, and the diagnostic electrode status algorithm issued the same warning for each event that could lead to the abnormal status.

## 5. Conclusions

In this study, an online measurement system was developed to identify the reliability and durability of a nitrate-selective electrode for sensing and measuring nitrate concentrations in real time. The proposed diagnostic algorithm can determine the electrode replacement time with the help of SDIs, which reflect the sensitivity change and signal drift of the electrode. The SDI method was effective in the laboratory-scale repetitive measurements; it signaled that an electrode needed to be replaced when the SDI reached 70. This SDI-based diagnosis algorithm was applied in an actual experiment; the accuracy of classification was 0.77.

The developed online wireless nitrate monitoring system and electrode diagnostic algorithm were used in a field application test. The monitoring and web-based alarm systems were successfully implemented, the electrodes were successfully replaced, and unidentified high-concentration events were detected. Hence, the developed monitoring system and electrode diagnostic algorithm can help with identifying the causes of water pollution.

## Figures and Tables

**Table 1:** Chemical compositions of NO_3_^−^ ISE membranes used in this study.

Component	Reagent	Composition
Ionophore	TDDA	4.0% (8 mg)
Plasticizer	NPOE	67.75% (135.5 mg)
Matrix	PVC	28.25% (56.5 mg)
Inner solution	0.01 M NaNO_3_ + 0.01 M NaCl	

**Table 2:** Confusion matrix for evaluation of diagnostic model.

		Actual Results	
		True	False
Model classification result	True	True Positive (TP)	False Positive (FP)
	False	False Negative (FN)	True Negative (TN)

**Table 3:** Actual performance characteristics of the SDI algorithm.

	SDI
Precision ($\pi_{i}$)	0.65
Recall ($\rho_{i}$)	0.87
Accuracy ($\alpha_{i}$)	0.77

## Data Availability

Not applicable.
